# Use of Symptoms Scores, Spirometry, and Other Pulmonary Function Testing for Asthma Monitoring

**DOI:** 10.3389/fped.2019.00054

**Published:** 2019-03-05

**Authors:** Marcella Gallucci, Paolo Carbonara, Angela Maria Grazia Pacilli, Emanuela di Palmo, Giampaolo Ricci, Stefano Nava

**Affiliations:** ^1^Department of Pediatrics, S. Orsola-Malpighi Hospital, University of Bologna, Bologna, Italy; ^2^Department of Specialistic, Diagnostic and Experimental Medicine (DIMES), University of Bologna, Alma Mater Studiorum, Bologna, Italy

**Keywords:** asthma, guidelines, asthma monitoring, lung function tests, children, adults

## Abstract

Asthma is a global problem affecting millions of people all over the world. Monitoring of asthma both in children and in adulthood is an indispensable tool for the optimal disease management and for the maintenance of clinical stability. To date, several resources are available to assess the asthma control, first is the monitoring of symptoms, both through periodic follow-up visits and through specific quality of life measures addressed to the patient in first person or to parents. Clinical monitoring is not always sufficient to predict the risk of future exacerbations, which is why further instrumental examinations are available including lung function tests, the assessment of bronchial hyper-reactivity and bronchial inflammation. All these tools may help in quantifying the future risk for each patient and therefore they potentially may change the natural history of asthmatic disease. The monitoring of asthma in children as in adults is certainly linked by many aspects, however the asthmatic child is a future asthmatic adult and it is precisely during childhood and adolescence that we should implement all the efforts and strategies to prevent the progression of the disease and the subsequent impairment of lung function. For these reasons, asthma monitoring plays a crucial role and must be particularly close and careful. In this paper, we evaluate several tools currently available for asthma monitoring, focusing on current recommendations emerging from various guidelines and especially on the differences between the monitoring in pediatric age and adulthood.

## Introduction

Asthma is one of the most widespread diseases in the world and affects about 300 million patients (1). Despite the high prevalence of asthma in industrialized countries, overall asthma control is still not completely satisfactory. In Europe, only 15% of patients under steroid treatment achieve adequate disease control (2, 3). These findings highlight the importance of optimizing the asthma management in order to improve disease control. Asthma monitoring is an essential step of disease management, which cannot disregard the understanding of the asthma pathogenesis. The main goals of an effective management is the achievement of an optimal control and the prevention of serious exacerbations and impairment of lung function. Achieving control is ensured by monitoring.

The tools for monitoring asthma include the assessment of frequency and severity of symptoms (patient/parents reported or through specific scores) and the use of objective measurements such as spirometry, airway hyper-responsiveness and inflammatory markers.

During the pediatric age, the asthma monitoring must take into account that children are “growing subjects” with the need for continuous treatment adjustments related to the different stages of development not only physical but also psycho-relational. Moreover, healthcare providers do not face a patient but a family unit that must take care of the problem, with all the implications related to school and sports activities.

All guidelines recommend periodic follow-up visits, the interval between visits depends on initial assessment and treatment. Despite the availability of several asthma guidelines, detailed recommendations on asthma monitoring in children and young people (aged 5–16) are poorly defined.

The 2007 American National Asthma Education and Prevention Program (NAEPP) guidelines focuses their attention on the concepts of “impairment and risk” to determine the levels of asthma control and severity, assessed through symptom frequency and measures of lung function among children 5–11 years of age (4). NAEPP guidelines introduced a categorization of asthmatic patients defining them as having “persistent asthma” if they have experienced two or more exacerbations treated with oral corticosteroids (OCS) in the previous 6 months, with a consequent increased future risk. More recently, GINA guidelines state that a previous severe exacerbation in the last 12 months, a history of access into an intensive care or intubation are major independent risk factors for exacerbations (4, 5).

Others factors that increase the future risk in children and adults include the following: socioeconomic or psychological problems, comorbidities such as obesity, chronic rhino-sinusitis, food allergy, exposure to smoke or allergens, low FEV1, higher bronchodilator reversibility, high SABA use, inadequate treatment, sputum or blood eosinophilia and elevated FeNO (5).

The latter two performed before and after the preventive therapy are useful in the assessment of response to medical treatments.

Therefore, the need to quantify the “future risk” for each patient, led the scientific community to search for specific indicators of future exacerbations, such as biomarkers, individual characteristics and genetic factors. These indicators could play a crucial role in asthma monitoring, although some methods are not yet standardized or used in clinical practice.

Scottish Intercollegiate Guidelines Network (SIGN) recommends the monitoring of asthma in children mainly through the assessment of symptoms, number of exacerbations, school absences, evaluation of therapy adherence and inhaler technique, measurement of height, and weight at least annually (6).

A close monitoring of asthma should include also an early detection of the impairment in lung function as well as the presence of bronchial hyper-responsiveness and bronchial inflammation. These findings are even more relevant in patients at risk, especially in those at high risk of exacerbations or with poor/inadequate disease control (7).

Ideally, a successful management should minimize both daily symptoms and risk factors for exacerbations/complications.

As recommended by the most recent published guidelines (GINA, NICE, SIGN/BTS) the follow up of asthmatic patients should be centered on continuing patients self-monitoring and periodical ambulatory visits for the assessments of the clinical status and lung function parameters (5, 6, 8).

The frequency of follow-up visits depends on asthma severity and the need of treatment adjustments. According to GINA recommendations, the lung function should be recorded at diagnosis, 3–6 months after starting treatment and “periodically” thereafter.

Among pediatric population, an adequate parents training can have a role in reducing the frequency of follow-up visits (9–11).

The main purpose of this article is to compare recommendations of various guidelines about asthma monitoring but also highlighting the differences between recommendations for pediatric and adult patients. In particular, we focus our attention on the availability of clinical tools for assessment of frequency and severity of symptoms, the use of lung function tests and inflammatory markers and the early detection of comorbidities and risk factors for severe asthma.

Monitoring asthma also means investigating the causes of poor medication adherence as well as comprising practical difficulties in using inhaler devices and understanding therapeutic plans especially in adult patients with comorbidities. The lack of awareness and detailed information regarding the importance of treatment adherence, avoidance of triggers, proper inhaler technique significantly contributes to the poor disease control.

Many tools are currently available for clinical and instrumental asthma monitoring, some of these can be performed by patients or caregivers (e.g., PEF), others (such as FeNO, spirometry, or asthma scores) can be made by general practitioners and/or by pulmonologists while some other (such as sputum analysis and oscillometry) only by pulmonologists.

In this paper, we will analyze each of these tools by evaluating different applications in the disease monitoring and comparing different guidelines.

## Clinical Tools

### Asthma Control Scores

The clinical history is crucial to assess the asthma control and should include simple key questions and specific asthma scores to collected information about exacerbations, limitations of daily activities, nocturnal awakenings and reliever medication use.

For young children, asthma control should be determined with help from the child's parent through.

Both in adult and children, GINA guidelines distinguishes between controlled, partly controlled and poorly controlled asthma based on level of symptoms during the past 4 weeks (5).

This categorization take into account the presence of daily symptoms (>2 per week), any night awakenings, reliever needed (>2 per week) and any activity limitation due to asthma, therefore, controlled asthma is defined by minimal daily symptoms and need of short acting bronchodilator, no nocturnal symptoms and no limitation of activities.

Further and more specific tools to assess the asthma control include quality-of-life measures such as questionnaires applicable in both adults and children (Table 1).

**Table 1:** Main Asthma control scores in children and adults.

	**Children**	**Adults**	**Normal value/Note**
Asthma Control Questionnaire (ACQ) (7 questions) (9)	Validated in adults and children older than 5 years		Well controlled ≤0.75, Inadequately controlled ≥1.5 Minimal important difference 0.5
ACQ shortened (5 five question symptoms only)	Validated in adults and children older than 5 years		More accurate for subjects with normal or near-normal FEV1
Asthma Control Test (ACT) (5 questions) (6)	Validated in children aged for 4–11 year olds	Validated in adults	Reasonably well controlled 20–24; under control 25
Mini Asthma Quality of Life Questionnaire (AQLQ) (32 questions) (10)	_	Validated in adults	Symptoms assessed over the preceding 2 weeks
Pediatric Asthma Quality of Life Questionnaire (PAQLQ) (23 questions) (12, 13)	Validated for age range 7–17 years	_	Higher scores indicate better quality of life
Royal College of Physicians (RCP) (3 questions) (11)	Not validated in children	Not well validated in adults	Probably useful in day-to-day clinical practice
Asthma Therapy Assessment Questionnaire (ATAQ) (20-item) (14)	Mainly used in research	Not used in adults	Include 4 different domains on symptom control, behavior and attitude barriers, self-efficacy barriers, and communication gaps

The Asthma Control Test (ACT) and the Asthma Control Questionnaire (ACQ) are recommended by all Guidelines and have been studied and extensively validated for both adults and children (the childhood ACT for children 4–11 years of age and the ACQ for children older than 5 years).

ACT includes 5 questions, 3 related to symptoms, 1 related to medication use, and 1 about overall control during the past 4 weeks with separate sections for parent and child; a score ≤ 19 indicate a poor symptoms control (15–17).

The C-ACT include seven items and it is divided into two parts. The first part is addressed to the child and consists of four questions on perception of asthma control, limitation of activities, coughing and awakenings at night. The second one is completed by parents and consists of three questions (daytime complaints, daytime wheezing and awakenings at night) with six response options. The score ranges from 0 (poorest asthma control) to 27 (optimal asthma control). ACT and ACQ are useful to assess the response to longer-term treatment.

The ACQ includes 7 questions, 5 related to symptoms, 1 on rescue treatment use and 1 on FEV1 finding; the control is assessed over the preceding week. For children with normal FEV1 a version of five-point questionnaire is preferable (12).

Other available scores include the Mini Asthma Quality of Life Questionnaire, validated for adults (its counterparts for patients 7–17 years of age is the Pediatric Quality of life Questionnaire) and the Royal College of Physicians 3 Questions (13, 14). The first values the control over the preceding 2 weeks and could be used to assess response to longer-term treatment trials. The latter although not well validated in both adults and children, could be used in day-to-day clinical practice thanks to his simplicity.

In the pediatric setting, GINA guidelines also include the Test for Respiratory and Asthma Control in Kids (TRACK) and the Composite Asthma Severity Index (CASI) both including the assessment of exacerbations. The TRACK is the first validated questionnaire designed to assess asthma control exclusively in young children (<4 years). This score may be more sensible in children since it reflects the changes in asthma control over a short follow up period and take into account the assessment of exacerbations (18, 19). Nevertheless, children may experience more exacerbations during one season vs. another while most of the clinical scores investigate trends over the last month, so they may not be totally indicative of asthma control in children with seasonal wheezing (20).

SIGN guidelines also recommend the use of the Pediatric Asthma Quality of life Questionnaire (PAQLQ) to assess health-related QoL in children with asthma and including 23 questions that investigate 4 domains (symptoms, activity limitations, emotional function, and environmental stimuli), validated for the age range 7–17 years (21, 22).

A further questionnaire is the Asthma Therapy Assessment Questionnaire (ATAQ), a 20-item parent-completed questionnaire, developed to assist clinicians to identify children at risk for adverse outcomes of asthma and including 4 different domains on symptom control, behavior and attitude barriers, self-efficacy barriers, and communication gaps (23).

A new score defined Severe Asthma questionnaire (SAQ) is being validated in adults; it can be used to detect the impact of both asthma symptoms and treatment on quality of life (24).

Usually, in daily practice, the possibility of using asthma control questionnaires and above all quality of life measures is significantly higher in the pediatric clinical routine.

Certainly, in the pediatric age the supervision of parents ensures a further control and makes these scores more reliable than those compiled by asthmatic patients. Moreover, in the adulthood comorbidities play an important role in the care management, often with reduced time to apply these clinical tools by healthcare professionals.

Concluding, we believe that asthma control scores are simple and useful monitoring tools, but the most of these refer to a short previous period and are often influenced by the subjective (or caregivers') symptom perception, for this reason they should be combined, when possible, with more objective tests such as pulmonary function tests or a careful clinical follow-up.

#### Patients and Parents Self-Monitoring

Patients as well as parents should be encouraged to keep track of symptoms consequently healthcare practitioners should adequately train them on this issue.

As recognized by several guidelines, many patients can benefit from a written action plan in which, according to the disease control, the patient is instructed to recognize the need for action (e.g., to step up therapy or seek medical advice) (5, 25).

A detailed education program for both adults and pediatric patients should cover: training on treatment adherence and correct use of medication, recognition and avoidance of triggers and risk factors for exacerbations or worsening of symptoms (such as exposure to allergens, influenza virus or rhinoviruses, smoke both active and passive or other environmental factors, including workplace related factors) (25, 26).

Among adults, educational programs have been repeatedly proven effective in improving symptoms control, quality of life and treatment compliance therefore they potentially can prevent or reduce severe exacerbations conducive to urgent visits and hospital admissions (4, 5).

A recent prospective randomized controlled trial including 160 adults with asthma showed that a single 10 min, educational session provided by a respiratory specialist, could substantially improve asthma control determined by the ACT score after 3 months. The educational program included basic information about asthma treatment and instructions on inhalation technique for about 10 min (27).

More recently, new tools for the self-assessment of asthma control are available such as applications for smartphones, often produced by respiratory societies, which can often be obtained for free (28, 29).

These applications enable patients to enter in their profile daily data such as symptoms and their frequency, ACT, PEF values etc. The app can therefore calculate the level of asthma control. Some apps have up to date pollen maps and calendars, or have personalized acoustic memos to remind patients to take the inhaled therapy (30).

Vasbinder et al. in their randomized controlled trial e-MATIC (e-Monitoring of Asthma Therapy to Improve Compliance in children), proposed the use of the “real-time medication monitoring (RTMM)” for improving adherence to inhaled corticosteroids. The study failed to prove a significant improvement in asthma control, quality of life or asthma exacerbations with high costs in the intervention group, although RTMM with tailored SMS reminders improved adherence to ICS (31).

Nevertheless, e- devices may be precious tools for monitoring, especially in adolescence. Teenagers, in particular, may experience age-related difficulties as they accept responsibility for self-management from their parents; the negative impacts of asthma are largely preventable if adolescents engage in self-management behaviors, including symptom prevention as treatment adherence and trigger avoidance or symptom monitoring. It has been proved that the use of “asthma apps” can positively influence adolescents' self-management behaviors through increased self-observation, self-judgment and increased self-efficacy (32).

The availability of these new promising resources certainly opens up new possibilities in the management and monitoring of asthma, even though a recent Cochrane meta-analysis (including 21 studies in adults and children), concluded that tele-healthcare for asthma did not seems to improve QoL or reduce exacerbation rate in children (33). Therefore, further evidences and studies will be needed to routinely recommend the use of these tools in the clinical practice.

## Lung Function Tests and Inflammatory Markers

### Spirometry

The functional hallmark of asthma is a reversible airway obstruction and its detection is often required for the diagnosis of the disease. The severity of obstruction is a known risk factor for exacerbations, therefore functional monitoring is essential in order to achieve optimal control.

Moreover, severity of obstruction does not always correlate with symptoms: a significant bronchial obstruction may be present also in asymptomatic children and adults. It has been shown that children with chronic obstruction are less likely to perceive the symptom of dyspnea than children with an acute obstruction (34). For this reason, children with poor perception of chronic obstruction are at risk of developing severe exacerbations, associated with poor lung function. Therefore, a regular assessment of lung function is crucial.

The spirometry is the main test for detecting and measuring airway obstruction in children over 5 years old and adults and it has some precision for predicting future attacks.

Reference values of the lung function tests suggested by several guidelines are reported in Table 2.

**Table 2:** Positive test threshold of objective tests in children (aged 5 years and over) and adults.

	**NICE** **(****8****)**		**GINA** **(****5****)**		**SIGN** **(****6****)**	
	**Children**	**Adults**	**Children**	**Adults**	**Children**	**Adults**
Obstructive spirometry	FEV1/FVC ratio <70% (or below the lower limit of normal if this value is available)		FEV1/FVC ratio <0.90	FEV1/FVC ratio <0.75–0.80	FEV1/FVC ratio <70%	
Bronchodilator reversibility test	Improvement in FEV1 of 12% or more	Improvement in FEV1 of 12% or more and increase in volume of 200 ml or more	Improvement in FEV1 of 12% or more	Improvement in FEV1 of 12% or more and increase in volume of 200 ml or more	Improvement in FEV1 of 12% or more	Improvement in FEV1 of 12% or more and increase in volume of 200 ml or more^**^
Peak expiratory flow variability	Variability over 20%		> 13%^*^	> 10%^*^	Variability over 20%^***^	
FeNO	35 ppb or more	40 ppb or more	FENO >50 ppb has been associated with a good short-term response to ICS		35 ppb or more	40 ppb or more
**CHALLENGE TEST**						
Methacholine and histamine (both non-specific direct broncho-provocation tests)	n/a	PC20°: 8 mg/ml or less	n/a	Fall in FEV1 of 20% or more with standard dose from baseline	n/a	C20 8 mg/ml or less°
Mannitol	n/i	n/i	n/a	Fall in FEV1 of 15% or more from baseline, with standard dose	Fall in FEV1 15% or more at cumulative dose of 635 mg	
Exercise challenge	n/i	n/i	Fall in FEV1 of > 12% predicted, or PEF >15%	Fall in FEV1 of > 10% and >200 ml from baseline	n/i	n/i

*Comparison between the different guidelines. n/a, not applicable; n/i, not included*.

* *Calculated from twice-daily readings (best of each time): (HighestPEF-LowestPEF)/mean of the day's highest and lowest PEF, and averaged over 1–2 weeks*.

** *School children using a threshold of 9% change*.

*** *Monitor peak flows for 2–4 weeks. °PC20: provocative concentration of methacholine causing a 20% fall in FEV1*.

The presence of expiratory airflow limitation should be valued at diagnosis or at the beginning of treatment (in order to evaluate increase in treatment dose), after 3–6 months of controller therapy and then periodically depending on clinical course, although SIGN and GINA guidelines do not indicate clear recommendations on monitoring FEV1 in children (5, 6).

NICE guidelines specify to perform a spirometry for monitoring asthma at each visit or at least after 3 or 6 months from the beginning of therapy and then every 1–2 years (8).

Spirometer parameters should be adjusted according to sex, age, and ethnicity. According to GINA guidelines, the FEV/FVC ratio cut-off of normality is 0.90 in children and 0.75–0.80 in adults (5). Different guidelines often diverge in the choice of this cut-off (according to NICE guidelines, it is 0.70 in both pediatric and adult age) (8) (Table 2).

In general, a fixed threshold might lead to an overestimation of obstruction in elderly patients and an underestimation in young ones (35).

In Table 3 we reported the main lung function tests used in our clinical setting in monitoring asthma.

**Table 3:** Main lung function tests used in our clinical setting in monitoring asthma.

	**Children >5 years**	**Adults**	**Normal value**	**Note**
Spirometry	Values widely available, usually within normal range in adults and children with asthma		FEV1/FVC >0.90 in children >0.75–0.80 in adults	Less applicable in acute severe asthma
Positive bronchodilator (BD) reversibility test from baseline suggestive for asthma	++	++	Children FEV1 <10% Adults FEV1 <12% and <200 mL	In children sometimes also suggestive also FEV1 >10% In adults more robust FEV1>15% and >400 mL
Peak expiratory flow (PEF) average diurnal variability over 2 weeks	Not routinely used	+	<8% with twice daily readings	Confirmed airflow limitation by variability
**AIRWAY RESPONSIVENESS**				
Exercise test	Used preferentially for diagnosis and not to monitor disease		Children <12-% adults <10%	Not applicable in patients with impaired lung function (i.e., FEV1/FVC <0.7 and FEV1 <70% predicted)
Exhaled nitric oxide (FENO)	Used only in specific protocols for diagnosis and monitoring		<25 ppb at exhaled flow of 50 ml/sec	>50 ppb highly predictive of eosinophilic airway inflammation and positive response to corticosteroid therapy
Eosinophil differential count in induced sputum	–	+	Normal range <2%	Close relationship between raised sputum eosinophil count and corticosteroid responsiveness

*+, dubious role in asthma monitoring; ++, potentially useful in asthma monitoring*.

FEV1 is the most widely used functional index in the asthma follow up; in particular, among asthmatic children a FEV1 <60% is a risk factor for exacerbations and its decrease is associated with increasing asthma severity (36). Children with FEV1 <60% of the predicted seem to have a double risk of asthma exacerbations in the following year compared to those with FEV1> 80% (4, 37).

In order to compare spirometry findings in children, Global Lung Initiative recommend that the spirometry values should be expressed in z score, even though these recommendation is poorly applied worldwide (5).

Adults with an accelerated FEV_1_ decline (>30 ml/year) may be either steroid-resistant/difficult-to-treat asthmatics or not adequately treated principally due to under-perception or poorly adherence to maintenance therapy (38).

Some authors have argued that other indices, such as FVC, should be also considered, as some patients with severe obstruction respond to bronchodilators with a significant increase in FVC but not FEV1 (39).

In order to compare spirometry findings in children, Global Lung Initiative recommend that the spirometry values should be expressed in z score, even though these recommendation is poorly applied worldwide (8).

It is well known that the confirmation of the diagnosis requires a positive reversibility test (according to GINA guidelines improvement in FEV1 ≥12 in children, ≥12% together with an increase in volume ≥200 ml in adults). American Thoracic Society recommendations define a significant bronchodilator response (BDR) as an increase in FEV1 ≥12% and/or 200 ml in both adults and children (40).

Some studies showed that in children, this cut-off may be too high and then less sensitive to assess airway obstruction, suggesting that a lower cut-off (8%) should be used to improve the diagnosis of asthma (41).

The assessment of bronchodilator reversibility (BDR) can be useful not only to confirm the diagnosis but also in the asthma monitoring. In severe pediatric asthma, the spirometry should be always performed with a bronchodilator test to detect airway obstruction and its reversibility since it has been shown that these children have an increased bronchodilator response that may be associated with higher risk of impairment of lung function (42, 43). A persistent BDR may also be associated with poor therapy compliance or wrong inhaler technique and seems to correlate to some indices of airway inflammation, such as the exhaled nitric oxide fraction (FeNO), therefore it might be predictive for a positive response to inhaled corticosteroids (ICSs) (44).

Regarding the most appropriate setting to perform lung function tests, although spirometry performed in the primary care setting may be a useful tool in asthma monitoring, concerns have been raised about the quality and standardization of this procedure compared to hospital-based or laboratory spirometry. The spirometry provides objective data of lung function, but the outcome is often dependent on the operator. Therefore, in our opinion, expert personnel that spurs the patient to an optimal execution should perform it.

### Peak Expiratory Flow

Home monitoring of peak expiratory flow (PEF) may be use as an additional functional test in the monitoring of asthma. There is still lack of evidence that PEF monitoring over time might result in better disease control. PEF measurement can be used to document the variability of bronchial obstruction in asthma even if PEF is not related to FEV1 values and may underestimate the degree of airflow limitation and air trapping. Moreover, PEF values vary depending on the meter used therefore it is advisable to compare its measurement with the best personal value (obtained during the disease control phase or during maximum treatment) using the same meter. PEF “personal best” has proved to be useful in improving the progression of asthma, but the patient needs to be adequately trained since measures are effort dependent (45, 46).

According with most of the guidelines, PEF measurement should not be routinely used to monitor asthma in children, unlike in adults where it is recommended for subjects with severe asthma or with poor perception of airflow limitation (5, 6, 8). Certainly, PEF measurements do not give information about the obstruction characteristics (obstructive or restrictive) or site. Nevertheless, NICE guidelines recommend a monitoring of peak flow variability for 2–4 weeks in children and young people (aged 5–16) if there is “diagnostic uncertainty after initial assessment with a normal or obstructive spirometry, irreversible airways obstruction (negative BDR) and a FeNO level of 35 ppb or more” (8). NICE guideline also recommend considering a value of more than 20% variability as a positive test. Even GINA guidelines in the diagnostic assessment of asthma include the use of diurnal PEF variability calculated from twice daily over 2 weeks; for children diurnal variability >13% is considered excessive [unlike in adult where the cut-off of PEF variability is >10% (5)] (Table 2).

The ease of execution even in pediatric age and the possibility of being performed at home and during acute phase make this test easy to handle and reproducible, even though its monitoring does not improve asthma control in addition to clinical scores in adults and children (6, 47). For this reason, PEF assessment is not recommended in pediatric age in asthma monitoring.

### Impulse Oscillometry, Forced Oscillations Technique, and Expiratory Flow Limitation

During childhood, impulse oscillometry (IOS) and the technique of forced oscillations (FOT) may be used as an alternative technique to assess lung function, since measurements are made from tidal breathing and younger children are able to comply compared to spirometry. IOS measures respiratory resistance and reactance by analyzing responses to pressure waves of different frequency. The assessment of airflow resistance can be an indirect indicator of airway caliber, while spirometry mainly reflects airflow characteristics. IOS is easily performed during tidal breathing therefore it only need a partial collaboration of small patients, even though it is not available in all centers and in some cases, it is difficult to interpret. ERS/ATS guidelines give practice information about the test modality and its analysis (48).

Several studies showed a significant association between findings of the IOS and those of spirometry. In asthma, IOS has been used to assess the bronchodilator response and the therapeutic response to different treatments. In studies utilizing both IOS and spirometry, the first one has proved to be more useful than spirometry in early detection of asthmatic children from normal cohorts (49).

Many evidence showed that peripheral airways (PAW) in children as in adults are the initial site of inflammation and obstruction in asthmatic disease (50).

IOS can evaluate peripheral airways more accurately than spirometry identifying a PAW impairment before symptoms and spirometric abnormalities occur. For these reasons, it could be used to guide an early therapeutic approach to prevent clinical symptoms and further lung damage (51).

In adulthood, excluding patients with severe chronic asthma and marked airway obstruction, an expiratory flow limitation (ELF) at rest is seldom observed, unless under severe and prolonged bronchoconstriction.

One way to value EFL is by the forced oscillation technique (FOT) through the application of negative pressure at the mouth during tidal expiration (NEP).

When the oscillatory pressure applied at the mouth does not reach alveoli during expiration due to a flow-limiting segment in the bronchial tree, the reactance signal, instead of reflecting the mechanical properties of the lung parenchyma and airways, is influenced only by those of the airways and becomes much more negative with a clear distinction between inspiration and expiration.

This application of the FOT is useful to identify flow limitation during tidal breathing, but the closure of intrathoracic airways eventually occurring at end expiratory lung volume (EELV) must be considered as an important limiting factor of this technique, since the distortion of the reactance signal is similar (52, 53).

In addition, when EFL originates in the peripheral airways, it is mainly due to the viscous, density-independent, flow-limiting mechanism, while the speed wave, density-dependent, flow-limiting mechanism is substantially involved when the EFL originates in the central airways.

Despite several potential applications of FOT and oscillometry, larger longitudinal studies will be needed to confirm the usefulness of these techniques as routinely monitoring tools in asthma.

### Blood and Sputum Eosinophils

As indicated in the recent ERS/ATS guidelines, the assessment of asthma phenotype (eosinophilic or non-eosinophilic) may play a crucial role in the management of patients with severe disease (54). The ideal tool for this purpose is represented by the cell count on BAL during bronchoscopy. The invasive nature of the procedure has obviously limited the number of subjects studied, therefore the scientific community has sought surrogates that allowed the identification of different asthma phenotypes such as eosinophils count in induced sputum and the peripheral eosinophilia.

Based on the sputum analysis, patients with asthma can be grouped in four different inflammatory phenotypes: eosinophilic asthma, neutrophilic asthma, mixed granulocytic asthma, and paucigranulocytic asthma. Eosinophilic asthma defined as a sputum eosinophil count of 2–3% or higher, represents almost half of the asthmatic population (55).

Several studies have found higher levels of sputum eosinophils in uncontrolled asthmatics, therefore sputum analysis may be a useful method of objectively monitoring asthma (56). Moreover, the short-term response to inhaled corticosteroids depends on the amount of eosinophils present in the sputum therefore this technique may be a guide for modulating steroid therapy (57).

A recent study by Fleming et al. included 55 children with severe asthma and showed that incorporating the control of sputum eosinophils into the management algorithm reduce exacerbations in the short term even though did not significantly reduce overall exacerbations or improve asthma control (58).

It is difficult for children to collect sputum because they tend to swallow more than expectorate.

Among pediatric patients with bronchial hyperactivity, induced sputum, through stimulation with hypertonic saline, may allow to understand the type of inflammation, the presence of cells and lower respiratory tract mediators (59–61).

Several studies have evaluated the safety of sputum induced in asthmatic children aged 6 to 16, demonstrating how moderate bronchospasm occurs in 10% of children and resolves with the administration of the bronchodilator (62, 63).

In clinical practice, the use of these tools for the diagnosis and monitoring of asthma certainly has limitations, however among pulmonologists and also in our center these may be a precious help for the assessment of the type of inflammation, the diagnostic confirmation and the adjustment of the preventive therapy.

In asthma, blood eosinophil are considered a good surrogate marker for sputum eosinophil count (over 2–3% with a cut-off of 220 cells per mm^3^ or 3% among adults). High eosinophil count in peripheral blood is a recognized risk factor for disease severity and for future exacerbations (58, 64). During childhood, the asthma predictive index (API) also include blood eosinophils within minor criteria as predictor of future recurrent wheezing (65, 66).

Nadif et al. showed that patients with high blood eosinophilia (>250 cells per mm^3^) had lower FEV1 values and worse asthma control than those with eosinophils in normal range (67).

For these reasons, the bronchial and peripheral eosinophilia could be considered a potentially useful biomarker for the selection of patients who will respond to anti IL5 therapy, a monoclonal antibody used in patient older than 12 years with refractory eosinophilic asthma.

### Fractional Exhaled Nitric Oxide (FeNO)

As already mentioned, the detection of different asthma phenotypes guided the scientific community searching for specific biomarkers that could guide and improve the disease monitoring and the therapeutic approach. The monitoring of asthma should also include the determination of minimally invasive inflammatory markers.

Fractional exhaled nitric oxide (FeNO) measurement correlates with eosinophilic airway inflammation and therefore with the most common asthma endotype, independently of gender, and age. FeNO levels are higher in asthmatic children compared to non-asthmatic children and in one study values rose further during exacerbations and rapid decline after oral steroid treatment (68–70). British guidelines recognize that a FeNO <20 ppb in children under 12 years may have a role in identifying patients who can step down corticosteroid treatment (5). This relationship is lost in adults smokers (superior cut off in children >35 ppb, in adults >50 ppb) (6) (Table 3).

Agency for Healthcare Research and Quality (AHRQ), recently conducted a systematic review (including 175 studies) about the role of FeNO in the diagnosis, treatment and monitoring of asthma. Both in adults and in children FeNO results can predict which patients will respond to inhaled corticosteroid therapy, therefore the use of this marker in long-term managing of treatments can reduce the frequency of exacerbations. Moreover, the review showed that FeNO diagnostic accuracy was modestly better in steroid-naïve asthmatics, children and non-smokers than the overall population. Nevertheless, regarding the asthma monitoring in preschooler children authors concluded that there is insufficient evidence supporting the use of FeNO in this category for predicting a future diagnosis of asthma (69).

Two recent Cochrane reviews, including both pediatric and adulthood studies, showed that tailoring asthma medications based on FeNO levels decreased the frequency of asthma exacerbations but did not impact on day-to-day clinical symptoms or inhaled corticosteroid dose (71–73).

In conclusion, FeNO role in asthma management has not been concretely proven due to incomplete evidence therefore it is not routinely recommended in all patients, at least in monitoring, even though it may be useful in subjects who respond poorly to inhaled corticosteroids (73).

Nevertheless, the use of biomarkers as tools for phenotyping asthma and personalizing therapy is certainly attractive but it has not yet entered clinical practice.

## Airway Hyperresponsiveness

### Bronchial Provocation Tests

A hallmark feature of asthma is increased responsiveness of the airways to inhaled stimuli. The assessment of bronchial responsiveness through provocation tests can be useful for both research purposes and clinical practice. Bronchial provocation tests include the direct inhalation of different substances such as methacholine, histamine, mannitol, inhalation of allergens or the use of “stimuli” such as exercise, inhalation of cold air and hyperventilation with dry air (74).

Monitoring of bronchial hyper-responsiveness (BHR) is not routinely recommended in current guidelines, since its role is more typically confined to the diagnostic process.

However, some data seem to indicate a potential usefulness of BHR among asthmatic adults, as an indicator of exacerbation risk and inhaled corticosteroid response (75, 76).

Bronchial provocations tests are not usually performed in asthmatic children and several papers support this recommendation including one clinical trial (77). Nevertheless, BHR assessments could have a role in asthma monitoring among children with exercise limitations or with reduced perception of symptoms (78).

Within the pediatric population, the exercise test may be a precious tool for the evaluation of indirect BHR (79). A reduction in post-exercise FEV1 compared to the baseline is considered a sign of bronchial obstruction induced by exercise. GINA guidelines recognize that the exercise challenge may provide information about airway hyper-responsiveness but “only undertake a challenge if it is otherwise difficult to assess asthma control.” A positive exercise challenge for children is considered for a fall in FEV1 >12% of predicted or PEF >15% (for adult a fall in FEV1 >10% and >200 ml from baseline).

NICE guideline clearly recommend of “do not use challenge testing to monitor asthma control,” while SIGN group state that “regular monitoring of airway responsiveness not proven to improve asthma control in children” (6, 8) (Table 2).

## Comorbidities and risk factors

Both for adults and children, the detection of potentially modifiable risk factors for exacerbations may be useful in asthma monitoring and includes the exposure to specific allergens, smoking, high SABA use, poor adherence to therapy and incorrect inhaler technique. As already mentioned, GINA guidelines state that a previous sever exacerbation in last 12 months and a history of access into an intensive care or intubation are major independent risk factors for exacerbations (5).

Moreover, the asthma monitoring cannot be separated from an early identification and management of associated comorbidities (Table 4).

**Table 4:** Principal asthma comorbidities.

	**Children**	**Adults**	**Note**
Anxiety and depressive disorders	++	+	Especially during adolescence
Gastro-esophageal reflux disease	+	++	More common in adults, although empiric treatment of asymptomatic GERD in asthmatics does not seem useful (68)
Obesity	++	+++	Asthma is more difficult to control in obese patients
Food allergy/anaphylaxis	+	+	Food allergy is a rare trigger but the association with asthma is a risk factor for anaphylaxis
Allergic rhinitis/Sinusitis	+	++	Often coexist
Nasal polyps	–	++	Exacerbated by aspirin or NSAIDs
Pregnancy	–	+	Change asthma control
Perimenstrual asthma	±	++	Possible role during adolescence
Respiratory infections	+++	++	Often exacerbation factors
Tobacco smoking and environmental exposure	++	+++	Chronic mechanism
Cardiovascular diseases	–	+++	Frequent in elderly
Chronic pulmonary diseases	–	+++	Frequent in elderly

–*, not relevant; ±, some relevance; ++, relevant; +++, very relevant*.

This term defines factors and/or pathological conditions, which can coexist with asthma, contribute to its severity and to poor control.

Comorbidities are obviously more frequent in adults and may significantly complicate the management of asthma throughout all its stages, from diagnosis to treatment. All guidelines present this point as relevant in the workup of asthma. The most frequent comorbidities among adult population include upper airway diseases (rhinitis, chronic rhinosinusitis), obesity, COPD, gastro-esophageal reflux disease (GERD), bronchiectasis; in elderly patients, heart failure is very common.

From an epidemiological point of view, rhinitis, and rhinosinusitis are the most frequent comorbidities of asthma for all ages and the former seems to be associated with an increased risk of exacerbations (80). The presence of GERD is associated with worse asthma symptoms and poorer quality of life while obesity can worsen asthma by compromising lung function, inducing corticosteroid insensitivity and systemic inflammation (81–83).

All these conditions often exacerbate or simulate symptoms of asthma causing a poor response to treatment. For these reasons, it is essential to assess and carefully monitor these comorbidities also by implementing integrated care pathways.

Other conditions that may present with elevated blood eosinophilia and a clinical picture mimicking a severe refractory asthma, such as chronic eosinophilic pneumonia, Churg-Strauss syndrome, allergic bronchopulmonary aspergillosis (ABPA), should be taken into account during asthma monitoring (84).

Finally, a subset of adult patients (usually over 40 years of age) present a combination of both asthma and COPD features which is known as Asthma-COPD overlap syndrome (ACOS), likely resulting from different phenotypes of airway disease. They are often smokers, but may have allergies and a family or personal history of asthma with a not completely reversible airway obstruction (5).

Additional diagnostic findings include eosinophilic airway inflammation, a good response to corticosteroid therapy, and high concentrations of exhaled nitric oxide, which should be assessed in the monitoring of these patients (85). Since ACOS outcome is generally worse than asthma with higher treatment needs, the management should be especially careful therefore it might be advisable to refer these patients to specialized center.

## Asthma Action Plan

The asthma action plan helps asthmatic adults and/or caregivers recognize worsening asthma and gives clear instructions on what to do in response. Each asthmatic patient is different, so each action plan will be too.

An accurate action plan should cover every of these points:

- What medicines to take and when
- A list of potential triggers
- Early symptoms of flare-ups and what to do if they happen
- Know how to manage a full-blown flare-up
- When to get emergency care

The asthma action plan should be based on symptoms trend or peak expiratory flow (PEF) measurements and is individualized according to the pattern of the patient's disease. In children, symptom-based plans are preferred.

Inclusion of PEF measurements in the asthma action plan can be beneficial for adults with more severe or difficult-to-control asthma and those with poor symptoms perception. When PEF is used, the asthma action plan should be based on personal best rather than on predicted values.

Regular review of the asthma action plan is an important part of asthma monitoring since the level of asthma severity and control may change over time (86, 87).

## Conclusion

An ideal asthma monitoring should provide a personalized approach for each asthmatic patient.

The personalized asthma care should use specific monitoring tools for different patients, preferably with different fields. This assumption therefore represent a fundamental part of comprehensive asthma management.

In light of reported evidences, it may be noted that some advice in monitoring of asthma may apply to both adults and children.

1. The categorization of asthma based on disease severity, which is crucial for the therapeutic planning, require an accurate combination and evaluation of the clinical and functional status.
2. The assessment of risk factors for exacerbations and for severe asthma should be carefully investigated.
3. Moreover, the estimation of future risk allows identifying patients who require a closed follow-up in order to prevent acute exacerbations and to detect an early impairment of lung function.
4. The use of a written and simple action plan (clear indications on which drugs to take every day, how to spot if asthma's getting worse, and what to do if you have an asthma attack) for both adults and children is advisable and it should be reviewed during each control.
5. At each visit, the patient's adherence to the treatment and correct use of inhaler devices should be assessed and reviewed. For patients who monitor their PEF at home, is advisable to review periodically the correct use of the instrument.
6. Pediatricians should always remember that a careful monitoring of growth and possible side effects of therapy is essential among asthmatic children.
7. Self-management tests associated with monitoring tools for the assessment of pulmonary function and measures of airways inflammation must be appropriate for pediatric age.

Ideally, the follow-up of asthmatic patients should have as its objective a responsible self-monitoring associated with a periodic outpatient check of the clinical status and lung function findings.

As healthcare professionals, we should arouse awareness and self-management. Particularly among adolescents, we should implement a shared decision-making and find ways to connect with them effectively.

The use of new technologies in monitoring asthma is inevitable and may help us to provide great opportunities to monitor patients remotely and to improve the communication.

## Data Availability

All datasets generated for this study are included in the manuscript and the supplementary files.

## Author Contributions

MG and PC performed the literature review. AP, SN, and EdP contributed to data collection and interpretation of the literature data. GR coordinated the writing group. All authors critically reviewed the manuscript, read and approved the final version.

### Conflict of Interest Statement

The authors declare that the research was conducted in the absence of any commercial or financial relationships that could be construed as a potential conflict of interest.

## Abbreviations

NAEPP: American National Asthma Education and Prevention Program
BDR: bronchodilator reversibility
BHR: bronchial hyper-responsiveness
GINA: Global Initiative for Asthma
FeNO: fractional exhaled nitric oxide
IOS: impulse oscillometry
FOT: technique of forced oscillations
COPD: Chronic Obstructive Pulmonary Disease.
